# A study on volumetric change of mandibular condyles with osteoarthritis using cone-beam computed tomography

**DOI:** 10.1038/s41598-024-60404-z

**Published:** 2024-05-03

**Authors:** Chang-Ki Min, Kyoung-A Kim, Kyung-Eun Lee, Bong-Jik Suh, Won Jung

**Affiliations:** 1 Department of Oral and Maxillofacial Radiology, Institute of Oral Bioscience, School of Dentistry, Jeonbuk National University, Jeonju, South Korea; 2 Department of Oral Medicine, Institute of Oral Bioscience, School of Dentistry, Jeonbuk National University, Jeonju, South Korea; 3 Research Institute of Clinical Medicine of Jeonbuk National University - Biomedical Research Institute of Jeonbuk National University Hospital, Jeonju, South Korea

**Keywords:** Temporomandibular joint disorders, Osteoarthritis, Cone-Beam computed tomography, Diagnostic X-Ray radiology, Medical research, Orofacial pain, Cone-beam computed tomography

## Abstract

This study aimed to quantitatively assess three-dimensional changes in the mandibular condyle with osteoarthritis using cone-beam computed tomography (CBCT). Pre- and post-treatment CBCT images of temporomandibular joints (TMJs) from 66 patients were used to assess longitudinal changes in condylar volume within individual patients using 3D slicer software. Total volume difference (dV), net increase (dV + , bone deposition), and net decrease (dV− , bone resorption) after treatment were analyzed based on clinical and radiological factors. Condyles with surface erosion at their first visit showed significantly decreased volume after treatment compared to condyles without erosion (*p* < 0.05). Amounts of bone resorption and deposition were higher in condyles with surface erosion (both *p* < 0.01). In patients with condylar erosion, the presence of joint pain was associated with a decrease in condylar volume and an increase in net resorption (both *p* < 0.01). When both joint pain and condylar erosion were present, patients with parafunctional habits showed reduced condylar volume after treatment (*p* < 0.05). Condylar volume change after treatment was negatively correlated with the duration of pain relief (R = − 0.501, *p* < 0.05). These results indicate that condylar erosion and TMJ pain could be significant variables affecting TMJ volume changes after treatment. Establishing appropriate treatment strategies is crucial for managing condylar erosion and TMJ pain.

## Introduction

Degenerative joint diseases (DJD), also known as osteoarthritis of the temporomandibular joint (TMJ), is a critical subtype of temporomandibular disorders (TMDs) that causes irreversible destruction of the TMJ^1,2^. DJD can cause serious complications including irreversible condyle deformity, jaw asymmetry, and bony ankylosis of the TMJ. Clinical signs and symptoms of DJD appear similar to those of other TMD subtypes, including pain, joint noise, and TMJ dysfunction^3–5^. Mechanical overload of the TMJ is considered a primary cause of DJD. Degenerative changes in the mandibular condyle occur due to an imbalance between mechanical overload and joint capacity^6,7^. Various factors such as age, systemic disease, and hormonal factors can affect this change^4^. However, a standard model of disease progression remains elusive because a variety of variables can affect DJD.

Progression of DJD can result in various osseous changes in the condyle, such as flattening, osteophytes, sclerosis, and erosion. These osseous changes can irreversibly alter the TMJ, leading to malocclusion and skeletal facial deformity^8,9^. Therefore, it is important to evaluate and predict these irreversible osseous changes in advance. However, several factors make this difficult. First, there is a lack of study on longitudinal changes in the condyle affected by DJD. Some studies have analyzed average condylar morphology in normal TMJs and TMJs with degenerative changes using cone-beam computed tomography (CBCT)^10–13^. However, for prognosis prediction, longitudinal studies within individual patients are essential. Second, although recent studies attempted longitudinal analysis, they used qualitative methods rather than quantitative approaches^3,8,9,14^. Third, correlations between clinical symptoms and radiological findings are still controversial^15,16^. Degenerative changes in the condyle are also observed in healthy control groups without clinical signs^10^. Research that performs quantitative evaluation of longitudinal condylar osseous changes and their correlations with radiological and clinical findings is needed.

Therefore, this retrospective study aimed to analyze intra-patient longitudinal changes in the mandibular condyle associated with DJD before and after treatment. The analysis focused on quantifying these changes, specifically volume change of the mandibular condyle shown in CBCT images. In addition, amounts of bone resorption and bone deposition were measured separately using more detailed volumetric analysis. Clinical factors and radiological findings influencing these volume changes were also investigated.

## Results

**Figure 1:**
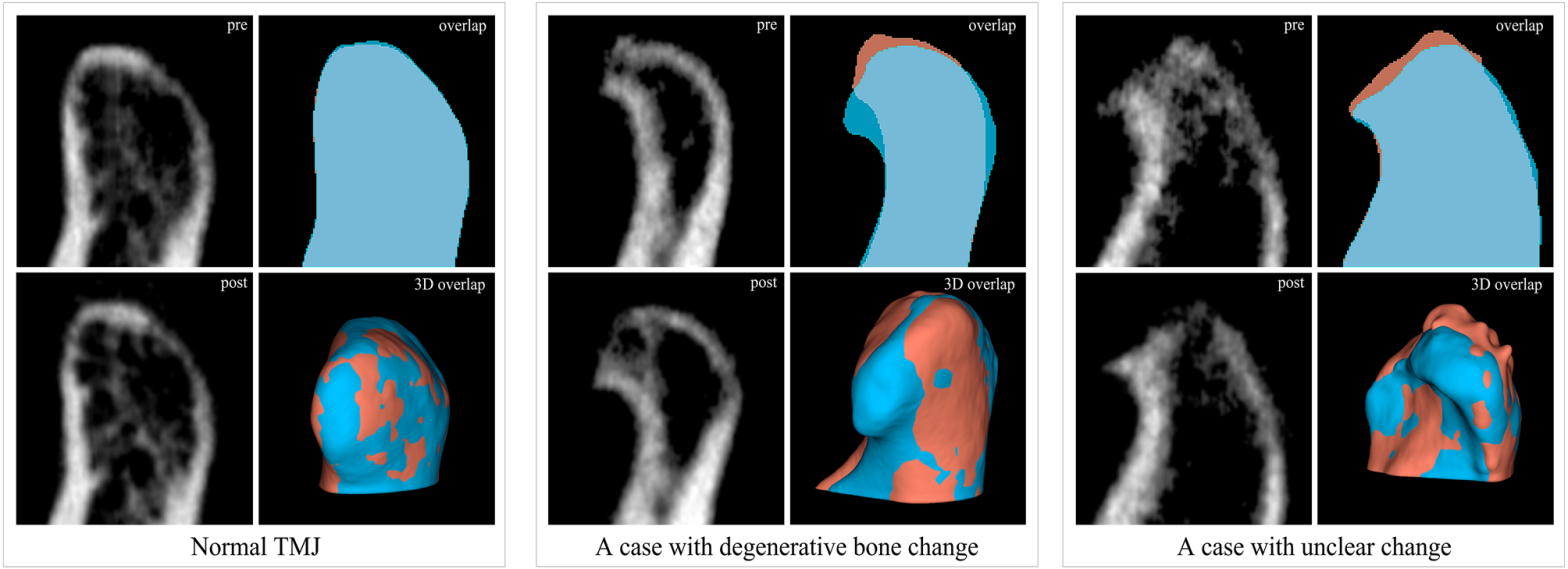
Representative images of temporomandibular joint (TMJ) cone-beam computed tomography (CBCT) images and image processing result. Sagittal CBCT images of pre-treatment (upper row) and post-treatment (lower row) condyle from a normal subject and patients with degenerative joint diseases. Overlap visualizes the comparison of segments between pre- and post-treatment condylar heads. Red segment, pre-treatment; Blue segment, post-treatment. 3D overlap shows rendered models of pre- and post-treatment condyle. Red segment, pre-treatment; Blue segment, post-treatment. The images were generated using the open-source software 3D Slicer (v4.11, Slicer, http://www.slicer.org/).

Demographic information associated with each participant is presented in Table 1. Representative results of image processing before and after treatment are shown in Fig. 1. More images of condyles are shown in Supplementary Fig. 1 and 2. For normal TMJs, pre- and post-treatment mandibular condyle almost completely overlapped. The volume difference between pre- and post-treatment (dV) of the normal TMJs was 8.01 ± 17.41 voxels out of a total condyle volume of 1318.191 ± 650.60 voxels, indicating minimal volume difference in the condyle. The volume difference in condylar neck and mandibular ramus of all study subjects after treatment was 20.82 ± 9.02 voxels out of a total of 1068.92 ± 486.92 voxels, showing the high accuracy of the segmentation and registration process of the study.

**Table 1:** Demographic information of study subjects.

Factors	Values
Age (years)^†^	44.21 ± 20.84
Sex (n, years)	
Male (10)	38.20 ± 22.37
Female (56)	42.29 ± 20.57
CBCT interval (months)^†^	14.18 ± 6.19

^†^Values are presented as mean and standard deviation. n, number of patients; CBCT, cone-beam computed tomography.

When pathological bone changes are present, parts of the condyle do not overlap each other. This is where bone resorption and bone deposition occur. (Fig. 1) As expected, bone resorption mainly occurred on the anterior superior joint surface. Bone deposition was observed in the periphery of the resorption site. Volumetric analysis was possible even in cases where the difference between pre- and post-treatment was not easily distinguishable on CBCT images with naked eye.

In the presence of surface erosion of the condyle, dV showed a significant (*p* < 0.05) decrease while the net increase (dV + , *p* < 0.001) and the net decrease (dV− , *p* < 0.01) were larger. (Table 2) Thus, condyles with erosion at the first visit experienced a more reduction in condyle volume after treatment compared to those without erosion, accompanied by more bone resorption and deposition.

**Table 2:** Comparison of various condylar volume changes between pre- and post-treatment based on the presence of erosion.

Factors	Yes (n = 90)	No (n = 42)	*P-*value
dV	− 51.64 ± 127.59	7.74 ± 127.59	0.012*
dV −	91.02 ± 122.96	13.41 ± 122.96	0.000*
dV +	40.20 ± 36.77	19.75 ± 36.77	0.001*

Values are presented as mean and standard deviation of voxels.

*, *p* < 0.05 in Mann–Whitney test.

Significantly smaller dV (*p* < 0.01) and larger dV−  (*p* < 0.01) were observed in the condyle when the patient had joint pain while dV + did not show a significant difference (Table 3). This indicates that the presence of joint pain is associated with greater bone resorption in the condyle with erosion. Table 4 compares volume changes according to the presence of various clinical factors in the condyle with erosion and pain. The group with parafunctional habits showed significantly smaller dV (*p* < 0.05).

**Table 3:** Comparison of condylar volume changes between pre- and post-treatment based on the presence of TMJ pain in condyles with erosion.

Factors	Yes (n = 65)	No (n = 25)	*P-*value
dV	− 73.96 ± 136.07	3.70 ± 82.58	0.004*
dV −	111.74 ± 135.54	39.64 ± 60.84	0.002*
dV +	39.02 ± 36.85	43.14 ± 36.98	0.722

Values are presented as mean and standard deviation.

*, *p* < 0.05 in Mann–Whitney test.

**Table 4:** Comparison of volume changes in condyle with both erosion and pain based on various clinical factors.

Factors	dV	dV −	dV +
Use of oral appliances			
Yes (38)	− 89.47 ± 159.83	134.93 ± 159.82	45.42 ± 40.32
No (7)	− 53.60 ± 97.16	93.54 ± 63.81	41.27 ± 32.01
*P* value	0.962	0.987	0.962
Stress			
Yes (11)	− 51.61 ± 149.83	115.54 ± 118.98	61.59 ± 54.53
No (34)	− 97.06 ± 152.59	135.68 ± 158.05	39.74 ± 32.31
*P* value	0.275	0.931	0.404
Parafunctional habits			
Yes (20)	− 137.14 ± 185.10	176.82 ± 195.14	41.13 ± 41.67
No (25)	− 39.29 ± 99.65	87.95 ± 81.55	47.78 ± 37.14
*P* value	0.040*	0.150	0.278
MOL			
Yes (28)	− 91.19 ± 170.05	141.55 ± 163.86	49.38 ± 39.83
No (17)	− 71.97 ± 119.42	107.37 ± 123.50	37.41 ± 37.42
*P* value	0.673	0.656	0.406
Missing posterior teeth			
Yes (2)	− 110.88 ± 49.19	135.32 ± 64.20	23.72 ± 13.19
No (43)	− 82.48 ± 154.59	128.01 ± 152.25	45.76 ± 39.44
*P* value	0.367	0.464	0.464
Change of occlusion			
Yes (11)	− 161.33 ± 209.59	206.42 ± 219.42	45.04 ± 42.34
No (34)	− 82.48 ± 119.42	128.01 ± 110.07	45.76 ± 38.30
*P* value	0.140	0.107	0.989

MOL, mouth opening limitation.

Values are presented as mean and standard deviation.

*, *p* < 0.05 in Mann–Whitney test.

Results of correlation analysis between volume changes and various factors are summarized in Table 5. Duration required for pain relief showed a significant positive correlation (*p* < 0.01) with dV− , while it showed a negative correlation with dV. The intensity of pain (NRS) on the first visit exhibited a significant negative correlation (*p* < 0.01) with dV +.

**Table 5:** Results of correlations between condylar volume measurements and clinical factors in condyles with both erosion and pain.

Factors	Pain duration	Age	CBCT interval (month)	Duration of pain relief (month)	Pain (NRS)	dV	dV −	dV +
Pain duration	1.000							
Age	0.180	1.000						
CBCT interval	− 0.053	0.027	1.000					
Duration of pain relief	− 0.045	0.188	0.422**	1.000				
Pain	− 0.79	− 0.098	0.005	0.208	1.000			
dV	0.179	− 0.043	− 0.136	− 0.501**	-0.229	1.000		
dV −	− 0.155	0.091	0.221	0.509**	0.143	− 0.963**	1.000	
dV +	0.052	0.136	0.316*	0.068	− 0.348*	0.165	0.105	1.000

NRS, numerical rating scale; pain duration, time to subjective pain relief after treatment began; dV, condylar volume difference between pre- and post-treatment; dV− , net decrease in condyle volume; dV + , net volume increase in condyle.

*, *p* < 0.05 and **, *p* < 0.01 in Spearman correlation analysis.

## Discussion

DJD is an irreversible condition. Predicting its prognosis is clinically important^4,5^. To predict condylar changes, quantitative analysis of longitudinal condylar changes is necessary. Studies assessing its association with clinical factors that influence these changes are also needed. Although several studies have assessed and analyzed condyles using CBCT images, many of them were cross-sectional, qualitative, or lacking consideration for clinical factors as described above^8,9,14–17^. Therefore, this study quantitatively evaluated longitudinal changes in condylar volume in patients with DJD after treatment.

Total volume change (dV) alone does not accurately reflect bone resorption in DJD because dV is the difference in value between pre- and post-treatment condyle volume. Bone resorption and deposition occur simultaneously during the progression of DJD, contributing to dV in opposite directions. Thus, the condylar morphology can change even without dV change when bone resorption and deposition occur at different sites. Therefore, we separately assessed amount of resorption (dV− ) and deposition (dV +) to better understand their changes. Results revealed significant differences not only in dV, but also in dV−  and dV + between groups with and without condylar erosion. In particular, it was noteworthy that not only bone resorption, but also bone deposition were increased in the condyles with erosion. This suggests that surface erosion reflects the state of active bone change accompanied by bone resorption and bone deposition. Some clinicians have questioned the need for intervention in asymptomatic condyles with erosion on CBCT because several studies have shown that DJD of TMJ is self-limiting^18,19^. These results indicated the need for treating asymptomatic condyles with erosion.

Among patients with surface erosion, significant differences in both dV and dV−  were observed according to the presence of TMJ pain, indicating more bone resorption. Additionally, there was a negative correlation between the subjective pain intensity and dV + value. This suggests that a higher pain intensity is associated with less condylar bone deposition. These findings imply the importance of TMJ pain as a crucial factor in predicting volumetric changes in the condyle after treatment. However, correlations between degenerative bone changes and TMJ pain remains controversial according to the previous studies^16,20,21^. This variability is because most studies are cross-sectional. Longitudinal analysis within intra-patient is considered to have higher sensitivity in evaluating bone changes than previous research. Therefore, a prospective study tracking the progression of osseous changes based on pain, including pain intensity and duration, is needed to validate results of this study in the future.

Pain duration and dV−  showed a positive correlation. It could be interpreted that bone resorption was more severe in patients who failed to control pain, or it could be interpreted that both pain duration and bone resorption were greater in patients who were not properly treated. Either way, it is clear that patients with successful pain control during the treatment have fewer pathologic bone resorption. For predicting prognosis following treatment, more research on the causal relationship between pain and bone resorption is needed.

Previous studies have shown that persistent mechanical overload of the TMJ can promote bone resorption. Clenching is a major cause of mechanical overloading. It is known that parafunctional habits can adversely affect the mandibular condyle^4,22^. However, the direct effect on volume change as shown in this study has been rarely reported. In the present study, patients with parafunctional habits showed a smaller dV, indicating reduction in condylar volume. Hence, patients undergoing treatment for DJD should be advised to control their clenching habits to minimize pathological bone changes.

In this study, MOL, loss of posterior teeth, and OSA treatment were analyzed as clinical variables. However, they did not show any statistically significant differences in condylar volume changes. The MOL in this study was a clinical variable that did not identify the cause of opening limitation. Degenerative bone changes in the condyle are inevitable after disc displacement without reduction (DDw/oR)^23^. The presence of DDw/oR might have influenced study results. It seemed that there was no significant result because MOL due to pain and MOL due to DDw/oR were combined. Apart from statistical results, loss of posterior teeth is considered meaningless as a variable due to a large difference in the number of participants between groups. Shetty et al. have reported a correlation between asymmetry in condyle volume and tooth loss^24^. However, the present study focusing on temporal changes in condyle volume within individuals did not reveal significant differences. OSA is a reliable treatment option for DJD^4,5,7^. Ok et al. have reported that OSA treatment has a positive effort on bone remodeling in the anterior part of the condyle in DJD^8^. However, in this study, OSA did not show significant differences in condylar volume changes. This was considered a limitation of the retrospective analysis. It is believed that the inability to control for variables other than OSA might have resulted in analysis not demonstrating significant differences. Further research is required to compensate for these limitations.

This study has several limitations. First, it was conducted retrospectively based on patients’ medical records. Because of constraints inherent in retrospective analysis, variables in the data were not controlled. Despite the drawback of reducing the sample size for controlling for data variables, we performed a stepwise analysis to refine the data. The small sample size was also a limitation. However, even with a small sample size, the power of test was found to be 0.83.

Second, the gray value of CBCT could be heterogeneous for the same subjects, unlike the Hounsfield unit of the CT. Gray values ​​of voxels in pre- and post-treatment CBCT images are also not completely homogeneous. To minimize this heterogeneity, only CBCTs scanned with the same equipment and under the same conditions were analyzed. Because volume change in the part not including the condylar head was very small, this heterogeneity did not appear to have a significant impact on study results. The reliability of auto-segmentation can also be an issue. Due to heterogeneity of the gray value, noise level, and ill-defined border of the erosion area, it was observed that the segment area changed drastically with a slight change in the threshold value. Therefore, even if the volume change in the area without bone change is very small, the measurement of change in the area with bone change may not be sensitive. Thus, significance might have existed even in analysis for which no significant differences were found in this study. This is a problem caused by the use of CBCT itself. It is difficult to completely resolve it. However, significant differences found in controlled conditions provide sufficient evidence.

Despite these limitations, this study provides a novel method for analyzing bone changes caused by DJD. Using this method, longitudinal bone changes were quantitatively analyzed over the course of treatment. Bone resorption and deposition were evaluated separately to obtain new information on disease progression. The presence of pain and erosion, and parafunctional habits were found to affect the amount of bone resorption and deposition that occurred during treatment. This study adds useful information to the current literature on correlations between bone changes and clinical/radiological findings observed during treatment for DJD. In the future, multi-center studies controlling for clinical variables are necessary to analyze volumetric changes in the condyle post-treatment. It is also necessary to analyze correlations between variables affecting volumetric changes and to study differences according to treatment strategy.

## Methods

### Subjects

Medical records and CBCT images of patients who visited the Department of Oral Medicine at Jeonbuk National University Dental Hospital between 2018 and 2020 with complaints of TMJ discomfort were reviewed.

Inclusion criteria were: (1) patients who underwent at least two CBCT scans of their TMJ by the same equipment, including their first visit; (2) patients who had been treated using conventional modalities including physical therapy, occlusal stabilization appliance (2 mm thick hard acrylic resin in the molar area; OSA), and medication; and (3) cases with consensus of the two experienced radiologists in the interpretation of the images. Exclusion criteria were as follows: (1) patients with other TMJ diseases such as condyle fracture, osteochondroma, osteomyelitis, rheumatoid arthritis, and systemic disorders affecting bone metabolism; (2) patients with facial trauma including TMJ; and (3) cases with blurry or poor CBCT images.

This study adhered to the Declaration of Helsinki. It was approved by the Institutional Review Board of the Jeonbuk National University Hospital (No. CUH2022-01–041). Informed consent was waived by the ethics committee (Institutional Review Board of the Jeonbuk National University Hospital) because of the retrospective nature of this study and the analysis using anonymous clinical data.

### Clinical information

Clinical variables were collected from patients’ medical records. Clinical information recorded at the first visit of each patient, including the presence and degree of TMJ pain, limited ability to open the mouth (MOL), loss of posterior teeth, occlusal changes, and clenching habits were reviewed. The degree of TMJ pain was evaluated using a numerical rating scale (NRS). MOL was defined as the case where the maximum mouth opening range was ≤ 40 mm. Follow-up CBCT was performed after clinical improvement in pain and TMJ function. The duration of pain during the subsequent treatment process and decision to use an oral appliance were also considered. The time interval between the two CBCT scans was recorded. Demographic information associated with each participant is presented in Table 1.

### CBCT scan

All CBCT scans were performed using an Alphard 3030 (Asahi Roentgen Ind., Co. Ltd., Kyoto, Japan) scanner with the following parameters: tube voltage, 80 kVp; tube current, 8 mA; FOV (field of view), 154 × 154 mm; and voxel size, 0.3 mm. Obtained images were reconstructed using the Ondemand3D software (CyberMed Inc., Seoul, Korea) for further analysis. Two TMJ scans, including those obtained during the first visit for each patient, were used to observe volume changes of the mandibular condyle in the same patient over time.

### Radiological diagnosis

Two oral and maxillofacial radiologists diagnosed TMJ CBCT images for surface erosion, subcortical cyst, or osteophyte formation according to the TMJ CT criteria presented by the DC/TMD. Subcortical cyst was defined as a cavity below the articular surface that deviated from normal marrow pattern Surface erosion was defined loss of continuity of articular cortex. Osteophyte formation was defined as marginal hypertrophy with sclerotic borders and exophytic angular formation of osseous tissue arising from the surface.^25^ Image diagnosis was confirmed by two experienced radiologists with a consensus.

### Image analysis

#### Image processing

**Figure 2:**
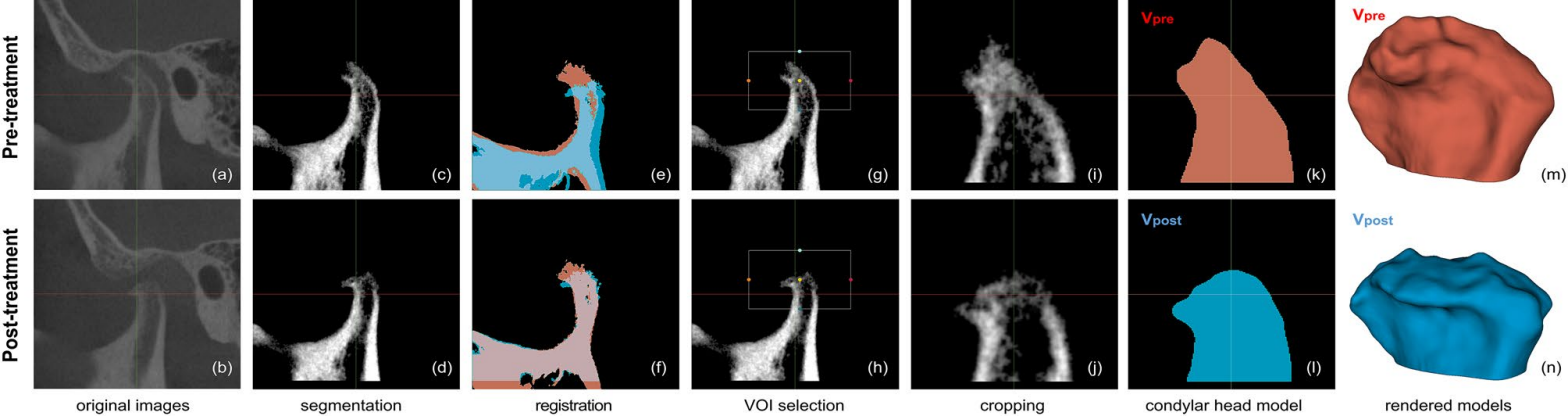
Image processing steps. (**a**–**b**) Original cone-beam computed tomography (CBCT) images of pre- and post-treatment from the same patient. (**c**–**d**) Pre- and post-treatment CBCT images after removal of cranial parts. (**e**–**f**) Alignment of pre- and post-treatment images by voxel-based registration and segmentation. Red segment, pre-treatment; Blue segment, post-treatment. (**g**–**h**) Selection of a common volume of interest (VOI) for both mandibles. (**i**–**j**) Cropped condylar head images by the common VOI. (**k**–**l**) Resultant segments of pre- and post-treatment condylar heads. (**m**–**n**) 3D model of each segment. Red, pre-treatment (Vpre); Blue, post-treatment (Vpost). The images were generated using the open-source software 3D Slicer (v4.11, Slicer, http://www.slicer.org/).

The open-source software 3D Slicer (v4.11, Slicer, http://www.slicer.org/) was used for volumetric analysis of a total of 132 CBCT images of condyles of 66 subjects^26^. Image processing steps are shown in Fig. 2.

#### Segmentation

Using automatic threshold function with maximum entropy method in the segment editor module, bony parts from pre-treatment CBCT were segmented. The obtained threshold value was saved for the segmentation of post-treatment TMJ CBCT of the same patient. CBCT of TMJ usually included cranial parts (such as the glenoid fossa and zygomatic arch) and mandibular parts (such as the mandibular condyle, mandibular ramus, and coronoid process). Among the segments, only the mandibular part was selected while others were removed. When segmenting the mandible from post-treatment TMJ CBCT images, we used the threshold value obtained from auto-thresholding pre-treatment CBCT of the same joint. Using these mandibular segments as a mask, only voxels corresponding to the mandible were cropped from the original CBCT. Median filter with radius of two voxels was applied to these cropped volumes to minimize noise.

#### Registration and cropping

To measure volume change after treatment, pre- and post-treatment mandibles were aligned at the same location using a voxel-based automatic registration function in the registration module. With two mandibles superimposed, a common volume of interest (VOI) containing only the condylar head part was set. Using this common VOI, each mandibular head part was cropped to obtain aligned condylar heads. These cropped condylar heads were used in the volumetric analysis described below.

To check accuracy of segmentation and registration, an additional VOI containing the condylar neck and mandibular ramus was set directly below the VOI of the condylar head. This was used on the premise that while the volume of condylar head changes as DJD progressed, other areas of the mandible showed little changes. Using additional VOIs, regions containing the condylar neck and mandibular ramus were also cropped and used for volumetric analysis along with the condylar head. The more accurate the segmentation and registration, the smaller this volume difference would appear.

#### Assessment of condyle volume change

Using the threshold value applied in the previous step, two cropped condylar heads were re-segmented to generate segment masks. Using the model maker function, surface models of masks were created and volumes of models were measured from each model information. Volumes of pre- and post-treatment condylar heads were designated as Vpre and Vpost, respectively. The value obtained by subtracting the Vpre from Vpost was named as dV, indicating total volume difference after treatment.

**Figure 3:**
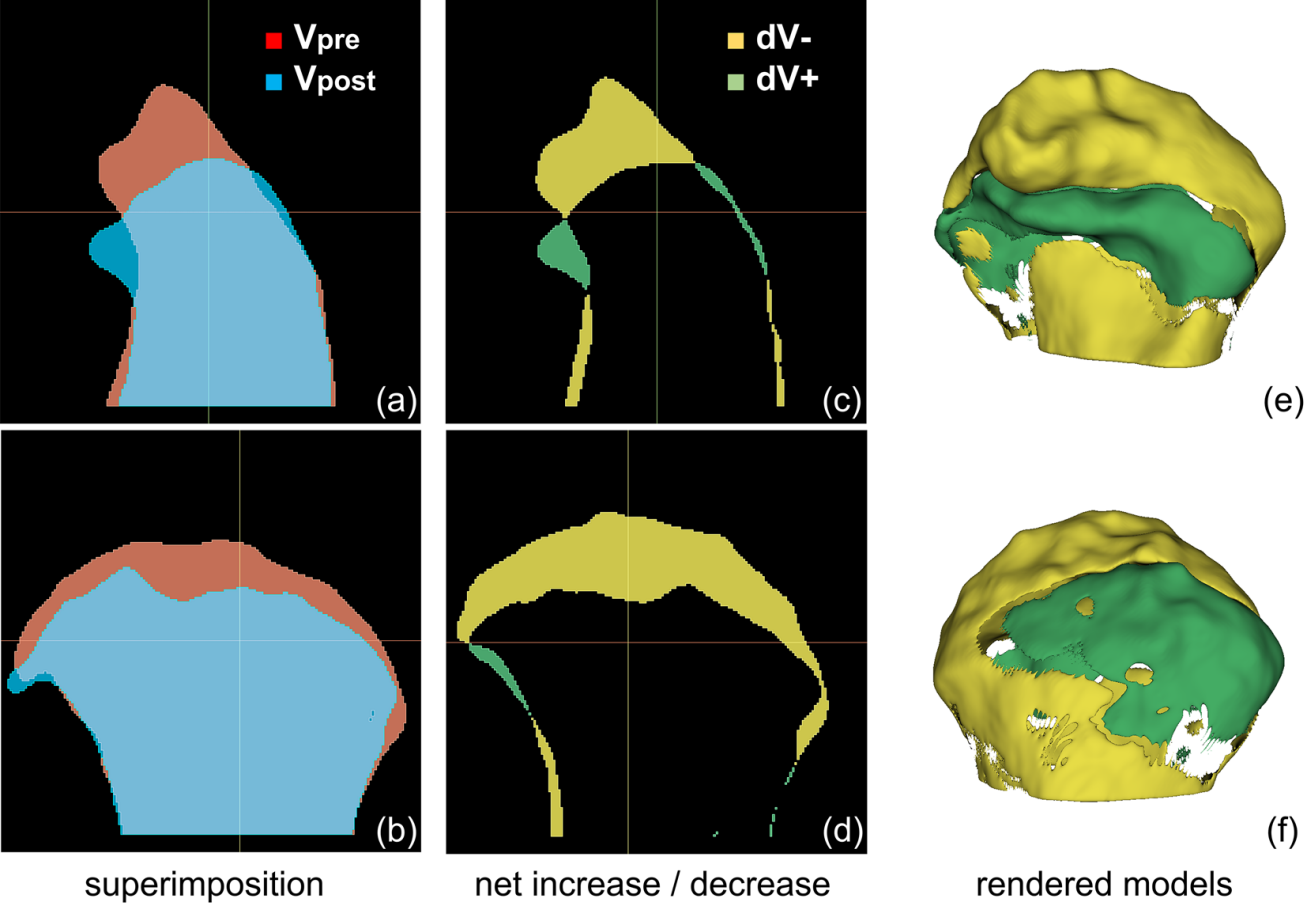
Representative volumetric analysis of processed cone-beam computed tomography (CBCT) images. (**a**–**b**) Superimposition of Vpre and Vpost in sagittal and coronal view. (**c**–**d**) Sagittal and coronal views of net decrease (dV− ) and net decrease (dV +). Yellow segment, dV−  = Vpre− overlap; Green segment, dV +  = Vpost-overlap. (**e**–**f**) Anterior and posterior views of the 3D model of dV−  and dV + . The images were generated using the open-source software 3D Slicer (v4.11, Slicer, http://www.slicer.org/).

To analyze detailed volume changes, net increase and net decrease of condylar volume were investigated. The volume obtained by subtracting the overlapping area from post-treatment segment was interpreted as a net increase (dV +) and the volume obtained by subtracting the overlapping area from pre-treatment segment was interpreted as a net decrease (dV− ). Names and interpretations of variables used in volumetric analysis are shown in Table 6 and a visualized example of this analysis is demonstrated in Fig. 3. Volume changes (dV, dV−  and dV) underwent further analysis as shown below.

**Table 6:** Parameters used in detailed analysis.

Parameter	Signification	Clinical interpretation
Vpre	Condylar head volume before treatment	
Vpost	Condylar head volume after treatment	
dV	Volume Vpost minus Vpre	
dT	Time interval between two CBCT scans	
dV−	Volume of Vpre minus overlapping area	Net decrease, bone resorption
dV +	Volume of Vpost minus overlapping area	Net increase, bone deposition

CBCT, cone-beam computed tomography.

### Data analysis

A total of 132 CBCT images of TMJ from 66 patients were included in this study. We analyzed differences in condyle volume changes (dV, dV− , dV +) between TMJs with and without surface erosion. Next, to investigate whether joint pain affected condyle volume changes when erosion was present, we compared condyle volume changes (dV, dV− , dV +) based on the presence or absence of TMJ pain using only 90 TMJ images with confirmed erosion.

We also investigated whether clinical factors influenced volume parameters. Cases with bilateral joint pain and surface erosion were excluded from this analysis because clinical factors were dependent on the patient, not the TMJ. We compared differences in volume parameters in 45 TMJ images based on the presence of various clinical factors.

### Statistical analysis

Mann–Whitney U and Kruskal–Wallis tests were used to assess effects of clinical and radiological factors on condylar volume change because some experimental groups did not satisfy normality in Shapiro-Wilks test. Spearman’s correlation analysis and multiple linear regression analysis were used to determine relationships between variables. Statistical significance was set at *p* < 0.05.

### Supplementary Information

Supplementary Information 1.

Supplementary Information 2.

Supplementary Information 3.

## Data Availability

The datasets used and analyzed in the current study are available from the corresponding author upon reasonable request.
