# Supplementary figures and images for: A study on volumetric change of mandibular condyles with osteoarthritis using cone-beam computed tomography
Source: Sci Rep. 2024 May 3;14:10232. doi: 10.1038/s41598-024-60404-z (PMC11068749; doi:10.1038/s41598-024-60404-z)

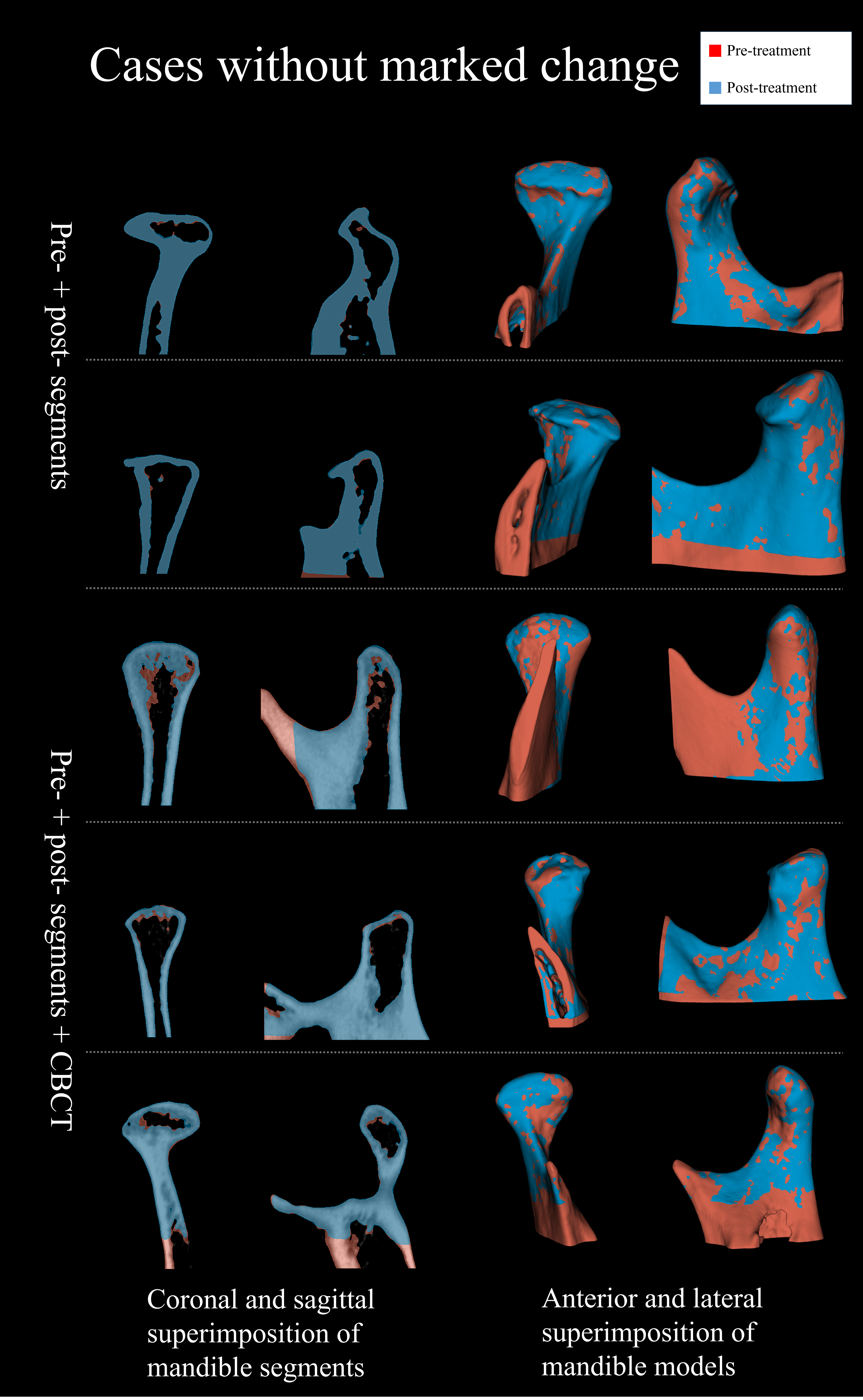

Supplement: Supplementary file 1 — Supplementary Information 1. [file 41598_2024_60404_MOESM1_ESM.tif]

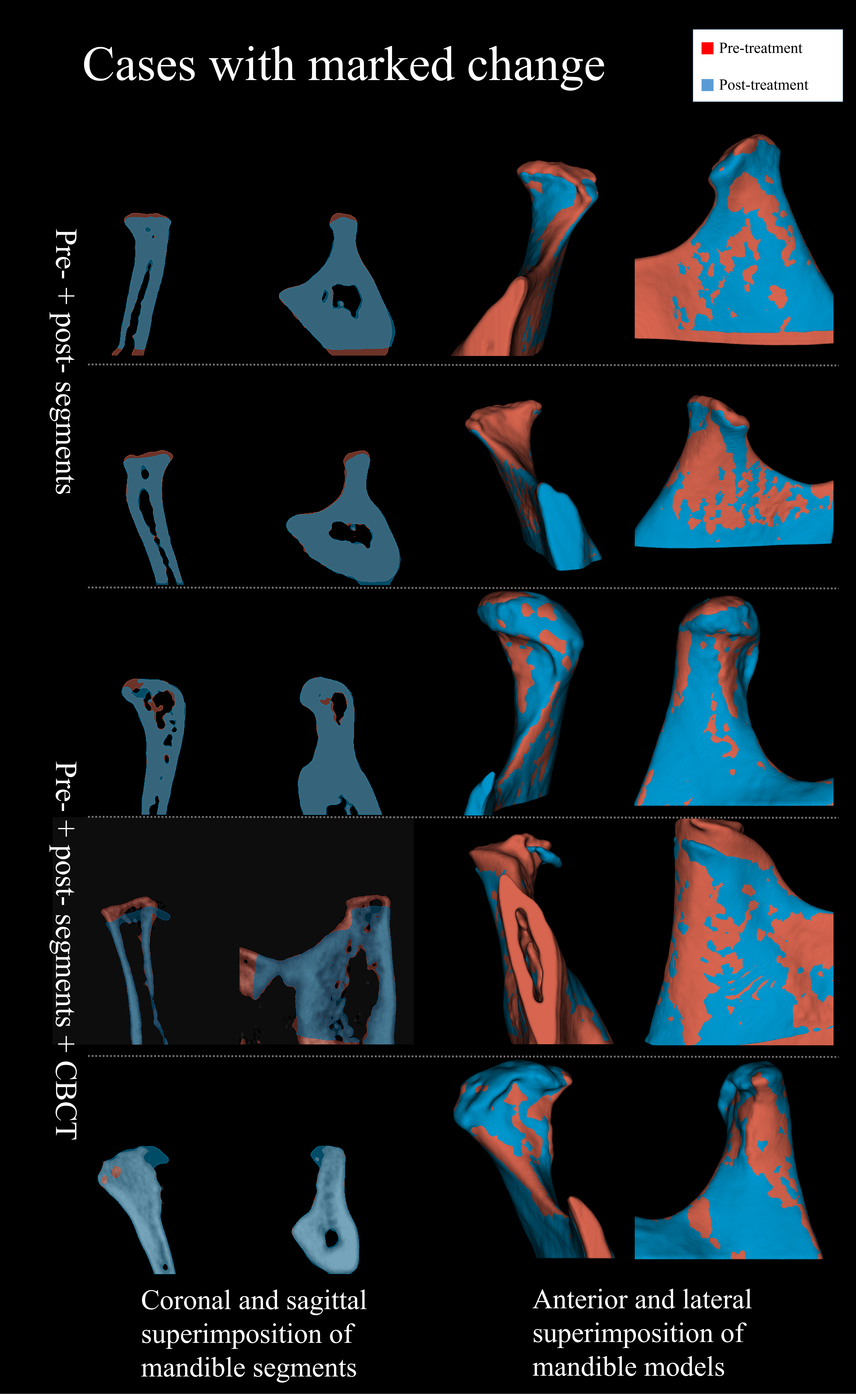

Supplement: Supplementary file 2 — Supplementary Information 2. [file 41598_2024_60404_MOESM2_ESM.tif]
