# Supplementary material for: A study on volumetric change of mandibular condyles with osteoarthritis using cone-beam computed tomography
Source: Sci Rep. 2024 May 3;14:10232. doi: 10.1038/s41598-024-60404-z (PMC11068749; doi:10.1038/s41598-024-60404-z)
Supplement: Supplementary file 3 — Supplementary Information 3. [file 41598_2024_60404_MOESM3_ESM.docx]

**Supplementary Figure 1. Segment superimposition of condyles without marked change.**

 The images demonstrate the superimposition of the pre- and post-treatment segmentations of the condyles after registration. Superimposition is shown in coronal and sagital view and superimposition in anterior and lateral aspects. It shows that there is little to no change in the condyle head after treatment. The images were generated using the open-source software 3D Slicer (v4.11, Slicer, <http://www.slicer.org/>).

**Supplementary Figure 2. Segment superimposition of consyles with marked change.**

The images demonstrate the superimposition of the pre- and post-treatment segmentations of the condyles after registration. Superimposition is shown in coronal and sagital view and superimposition in anterior and lateral aspects. It shows marked changes in the condyle head after treatment. The images were generated using the open-source software 3D Slicer (v4.11, Slicer, <http://www.slicer.org/>).
